# Author Correction: Stimulus-dependent representational drift in primary visual cortex

**DOI:** 10.1038/s41467-021-25825-8

**Published:** 2021-09-10

**Authors:** Tyler D. Marks, Michael J. Goard

**Affiliations:** 1 Neuroscience Research Institute, University of California, Santa Barbara, CA USA; 2 Department of Molecular, Cellular, and Developmental Biology, University of California, Santa Barbara, CA USA; 3 Department of Psychological & Brain Sciences, University of California, Santa Barbara, CA USA

**Keywords:** Neural circuits, Sensory processing

Correction to: *Nature Communications* 10.1038/s41467-021-25436-3, published online 27 August 2021.

The original version of this Article contained an error in a sentence in the Results section which incorrectly read ‘Repeating these analyses for noise correlations revealed a weaker effect than that of SC, though the trend was in the same direction (Supplementary Fig. 13), possibly due to relatively low levels of noise correlations in our data.’ The correct version refers to ‘Supplementary Fig. 14’ in place of ‘Supplementary Fig. 13’.

This has been corrected in both the PDF and HTML versions of the Article.

