# Supplementary material for: Stimulus-dependent representational drift in primary visual cortex
Source: Nat Commun. 2021 Aug 27;12:5169. doi: 10.1038/s41467-021-25436-3 (PMC8397766; doi:10.1038/s41467-021-25436-3)
Supplement: Supplementary file 1 — Supplementary Information [file 41467_2021_25436_MOESM1_ESM.pdf]

SUPPLEMENTARY INFORMATION

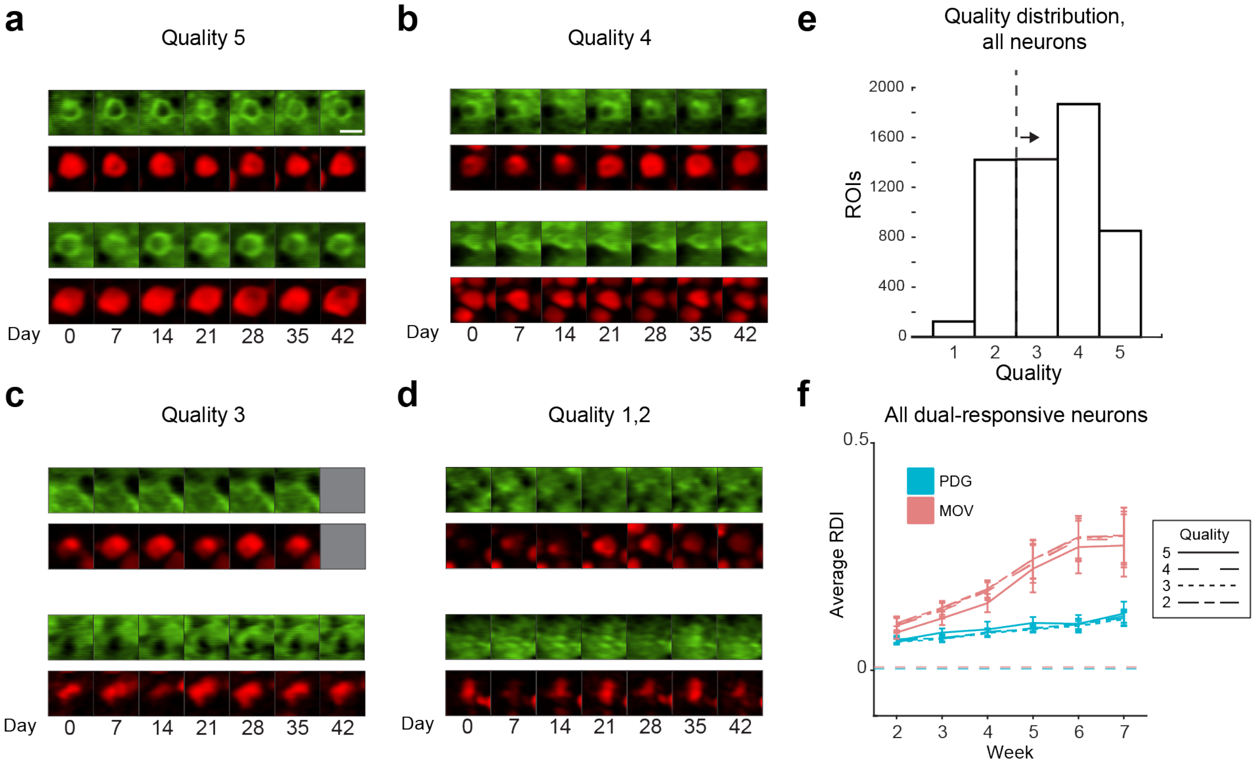

Supplementary Figure 1: Example neuron images and ROI quality distribution.

(a) - (d) Example ROIs from each quality rating. See methods for descriptions of each rating. Top row, green: average projection of GCaMP6s fluorescence channel. Bottom row, red: activity map of active pixels (see Methods). Scale bar = 15µm.

(e) Distribution of quality ratings. Data shown for all ROIs across all mice. For all analyses, ROIs of quality 2 or below are discarded.

(f) Average RDI curves across imaging fields for both stimuli as shown in Fig. 1i, for different ROI quality rating inclusion thresholds. Error bars are ± s.e.m. (quality threshold of 2: n = 956, 937, 920, 962, 890, 814 neurons from 13, 12, 12, 13, 11, 9 imaging fields for sessions 2-7 respectively; quality threshold of 3: n = 824, 808, 793, 830, 761, 698 neurons from 13, 12, 12, 13, 11, 9 imaging fields for sessions 2-7 respectively; quality threshold of 4: n = 624, 617, 592, 624, 581, 531 neurons from 13, 12, 12, 13, 11, 9 imaging fields for sessions 2-7 respectively; quality threshold of 5: n = 237, 234, 218, 236, 223, 208 neurons from 13, 12, 12, 13, 11, 9 imaging fields for sessions 2-7 respectively).

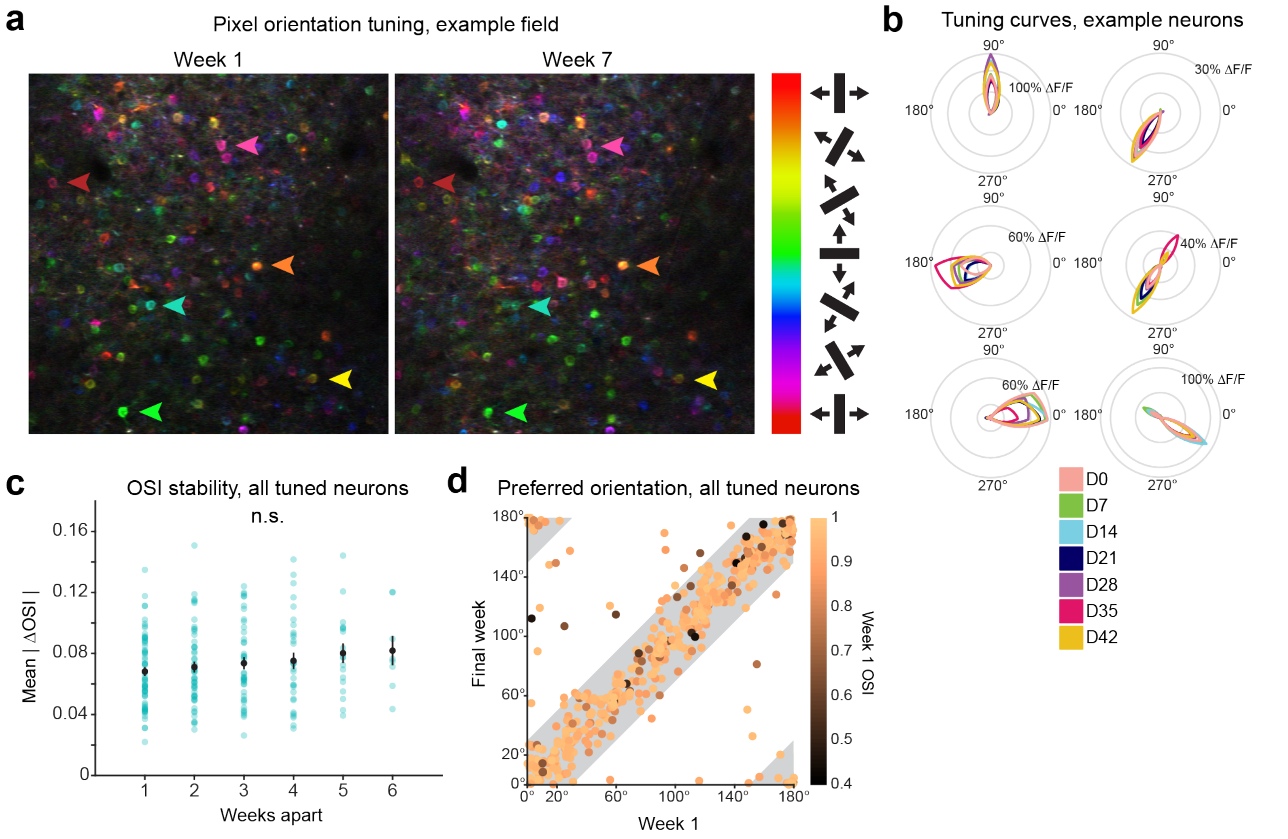

Supplementary Figure 2: Orientation tuning and selectivity are highly stable.

(a) Example PDG recording pseudocolored by pixel-wise preferred orientation on the first recording week and the seventh recording week. Arrowheads indicate a subset of example neurons that were well tracked across recordings.

(b) Tuning curves of example orientation-tuned neurons from one mouse across all recording sessions.

(c) Average change in orientation selectivity index (OSI) as a function of time between recordings. Data shown for all mice, neurons must be present on both sessions and orientation tuned on the first recording session to be included. Blue dots are individual pairwise comparisons between recordings, black dots indicate mean ± s.e.m. of each distribution (n = 66, 53, 43, 31, 18, 8 sessions for 1-6 weeks apart respectively). No timespans are significantly different from average mean |ΔOSI| (t_{65} = -1.4, p = 0.14, t_{52} = -0.4, p = 0.70, t_{42} = 0.3, p = 0.77, t_{30} = 0.5, p = 0.62, t_{17} = 1.2, p = 0.24, t_{7} = 1.0, p = 0.36 for 1-6 weeks apart respectively, p > 0.05 for all comparisons, two-tailed one-sample t-test).

(d) Preferred orientation on the first recording session vs. final recording session (see Methods). Data shown for all mice using neurons present on all sessions and orientation tuned on the first session (n = 603 neurons). Each dot is one neuron, colored by its OSI on week 1. Shaded areas indicate ± 30° change between recordings (± 1 orientation step, 96% of all neurons tuned on the first session).

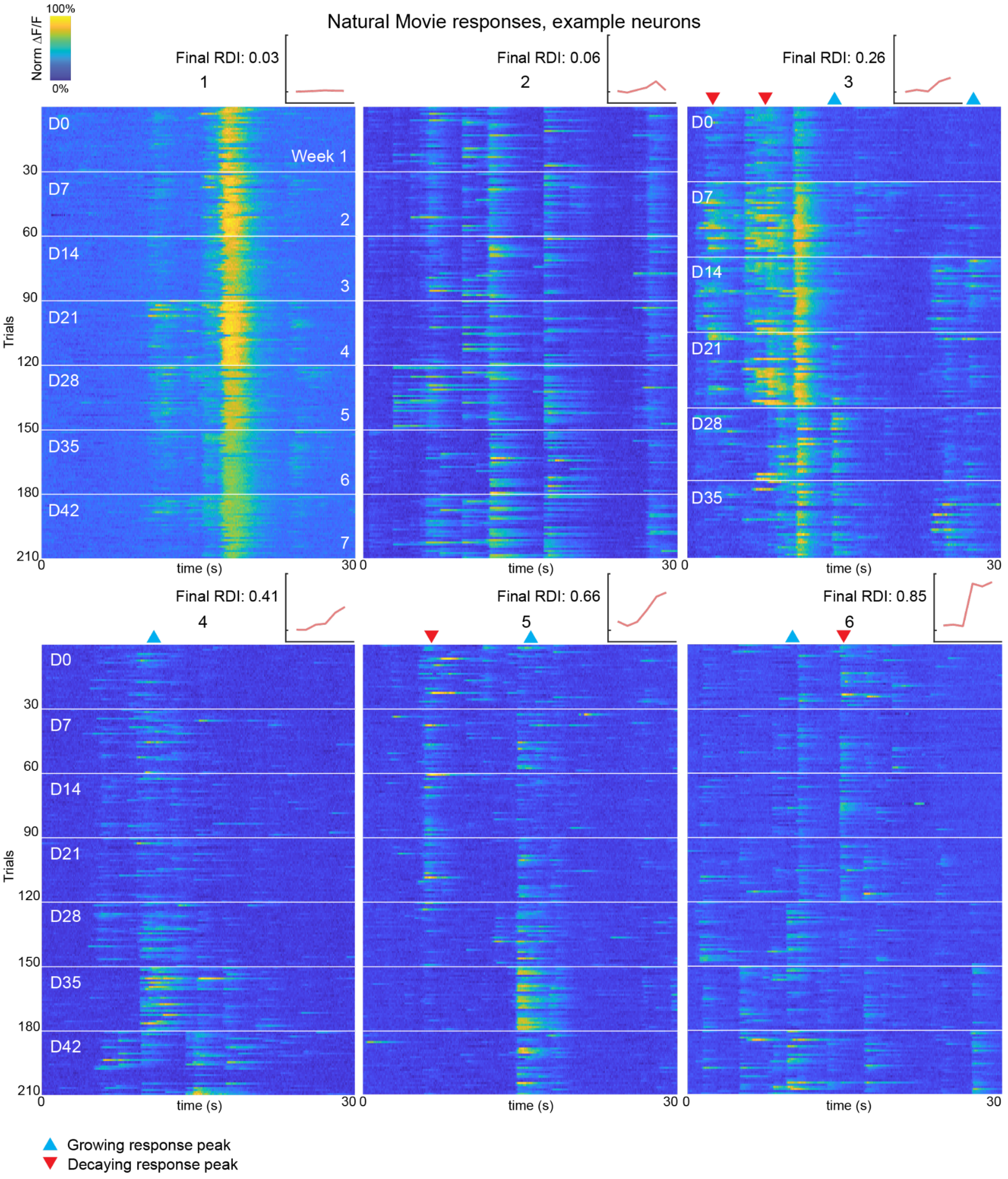

Supplementary Figure 3: Example natural movie responses.
Additional examples of single neuron responses to the MOV stimulus. White borders indicate the separation of recording sessions. Each neuron's MOV RDI curve is shown on the top right of each heatmap. Data is normalized across sessions.

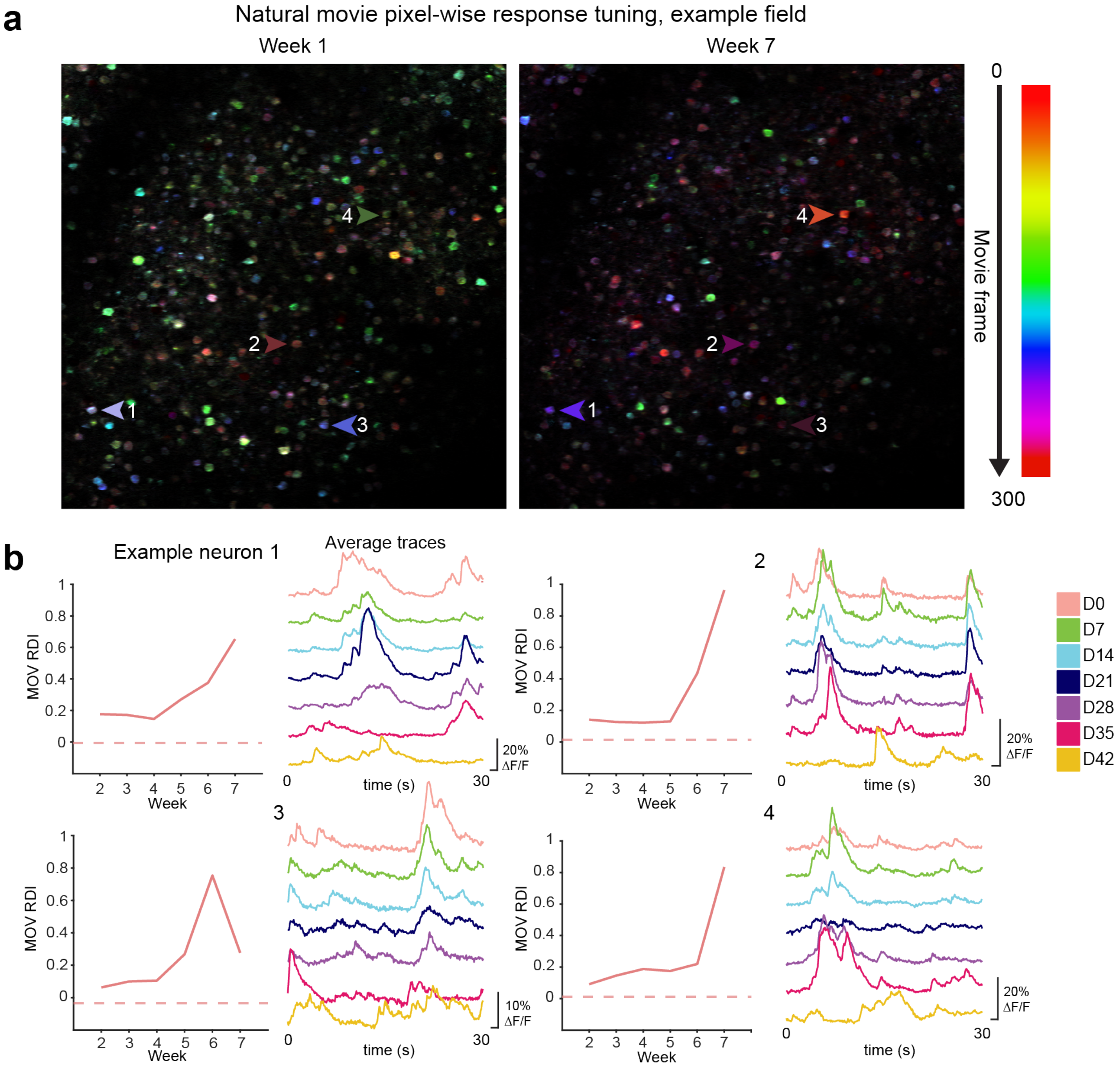

Supplementary Figure 4: Population responses to MOV stimulus.

(a) Pixels of an example MOV recording pseudo-colored by response timing on the first recording week and the seventh recording week. Arrowheads indicate a subset of example neurons that were well tracked across recordings.

(b) Example RDI curves and trial-averaged ΔF/F traces across sessions for neurons shown in (a).

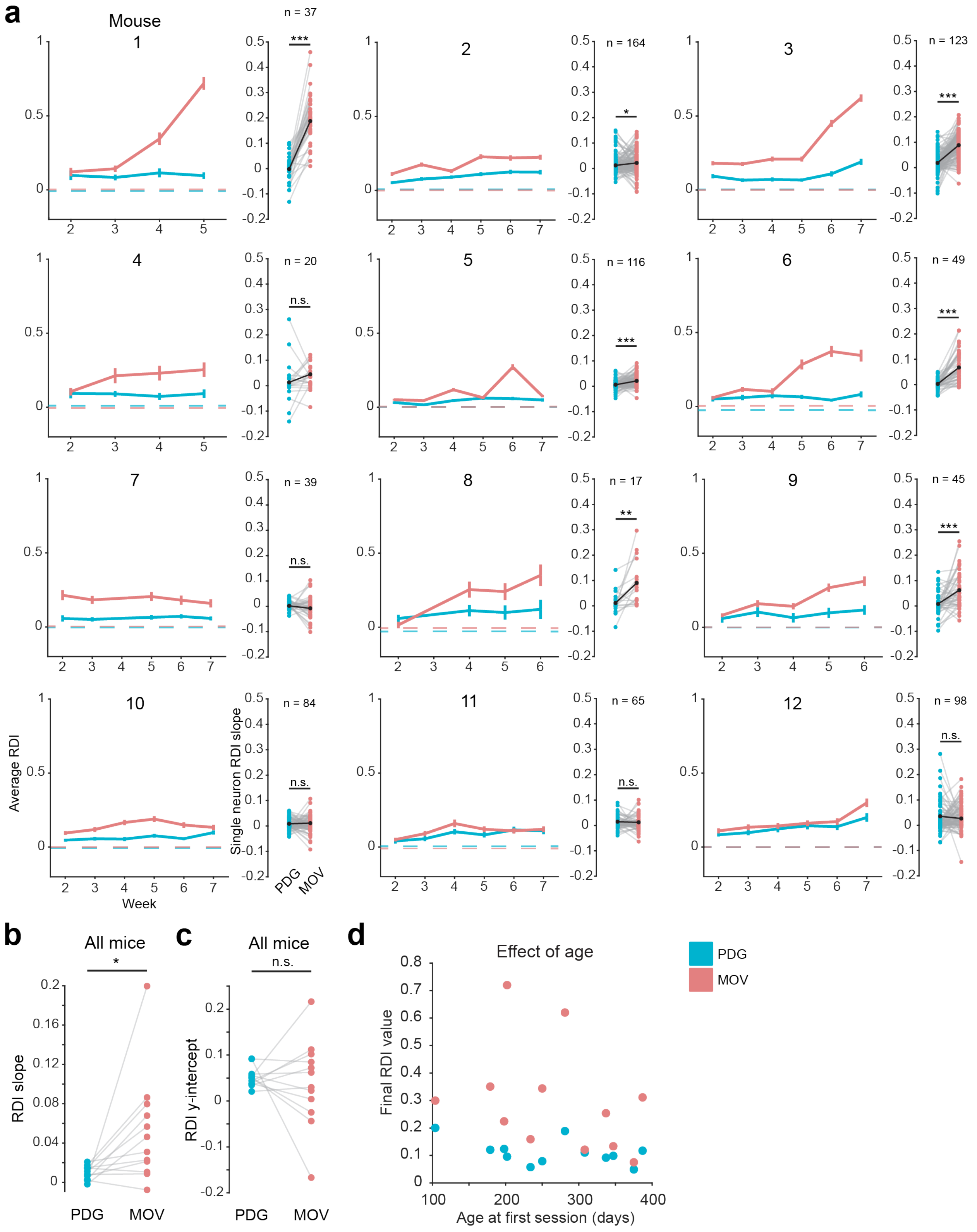
a
Mouse
1
2
3
4
5
6
7
8
9
10
11
12
n = 37
n = 164
n = 123
n = 20
n = 116
n = 49
n = 39
n = 17
n = 45
n = 84
n = 65
n = 98
***
*
***
n.s.
***
***
n.s.
**
***
n.s.
n.s.
n.s.
Average RDI
Single neuron RDI slope
Week
PDG
MOV
b
All mice
*
RDI slope
PDG
MOV
c
All mice
n.s.
RDI y-intercept
PDG
MOV
d
Effect of age
Final RDI value
Age at first session (days)
PDG
MOV

Supplementary Figure 5: Individual mice exhibit variability in amount of representational drift.

(a) Line plots: Average RDI curves for every imaged mouse. Values are calculated using only neurons that are present on any given session and responsive to both stimuli. Error bars are ± s.e.m.; dotted line indicates control RDI, as in previous figures. Scatter plots: pairwise comparison of RDI curve slopes for single neurons (*n* value is in neurons), each linked pair is one neuron (mouse 1: $t_{36} = 10.9$, $p = 5.7 \times 10^{-13}$, mouse 2: $t_{163} = 2.0$, $p = 0.047$, mouse 3: $t_{122} = 11.7$, $p = 1.0 \times 10^{-21}$, mouse 4: $t_{19} = 1.3$, $p = 0.22$, mouse 5: $t_{115} = 5.2$, $p = 1.1 \times 10^{-6}$, mouse 6: $t_{48} = 8.5$, $p = 4.0 \times 10^{-11}$, mouse 7: $t_{38} = 1.1$, $p = 0.30$, mouse 8: $t_{16} = 3.7$, $p = 0.002$, mouse 9: $t_{44} = 4.4$, $p = 6.6 \times 10^{-5}$, mouse 10: $t_{83} = 0.41$, $p = 0.68$, mouse 11: $t_{64} = 0.44$, $p = 66$, mouse 12: $t_{97} = 0.68$, $p = 0.50$; two-tailed paired-sample t-test; $^{*}p < 0.05$, $^{**}p < 0.01$, $^{***}p < 0.001$)

(b) Comparison of average RDI curve slopes for each stimulus across all mice. Each linked pair is one mouse ($n = 12$ mice, $t_{11} = 2.6$, $p = 0.025$, two-tailed paired-sample t-test; $^{*}p < 0.05$).

(c) Comparison of average RDI curve y-intercepts for each stimulus across all mice. Each linked pair is one mouse ($n = 12$ mice, $t = 0.44$, $p = 0.67$, two-tailed paired-sample t-test).

(d) Effect of mouse age on final RDI values for both stimuli (PDG Pearson $r = -0.46$, $p = 0.13$, MOV Pearson $r = -0.25$, $p = 0.27$).

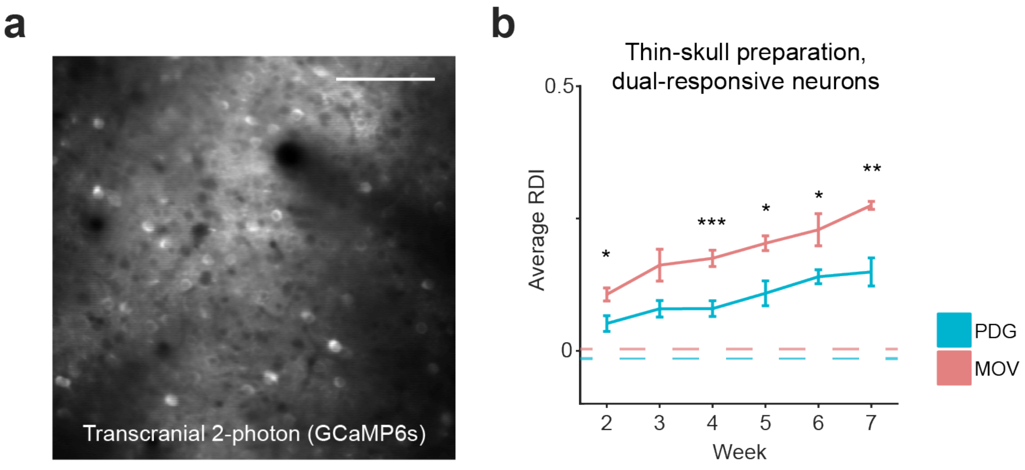

Supplementary Figure 6: Method of surgical preparation does not influence RDI curves
(a) Average fluorescence image from example transcranial imaging field. Scale bar = 100 µm.
(b) Average RDI curves for 3 mice (*n* = 81, 84, 84, 79, 75, 50 neurons in 3 fields for sessions 2-5 and 2 fields for session 6) imaged through a thin skull preparation instead of a cranial window. Error bars are ± s.e.m. Significance markers indicate the comparison of each session's PDG RDI values and MOV RDI values ($F_{1,160} = 5.5$, $p = 0.02$, $F_{1,166} = 3.7$, $p = 0.06$, $F_{1,166} = 17.3$, $p = 5.1 \times 10^{-5}$, $F_{1,156} = 6.1$, $p = 0.01$, $F_{1,148} = 5.4$, $p = 0.02$, $F_{1,98} = 8.1$, $p = 0.006$, for sessions 2-7 respectively; two-tailed F-test using a linear mixed-effects model, fixed effect for stimulus, random effect for mouse; *$p < 0.05$, **$p < 0.01$, ***$p < 0.001$). Dotted line indicates control RDI, as in previous figures.

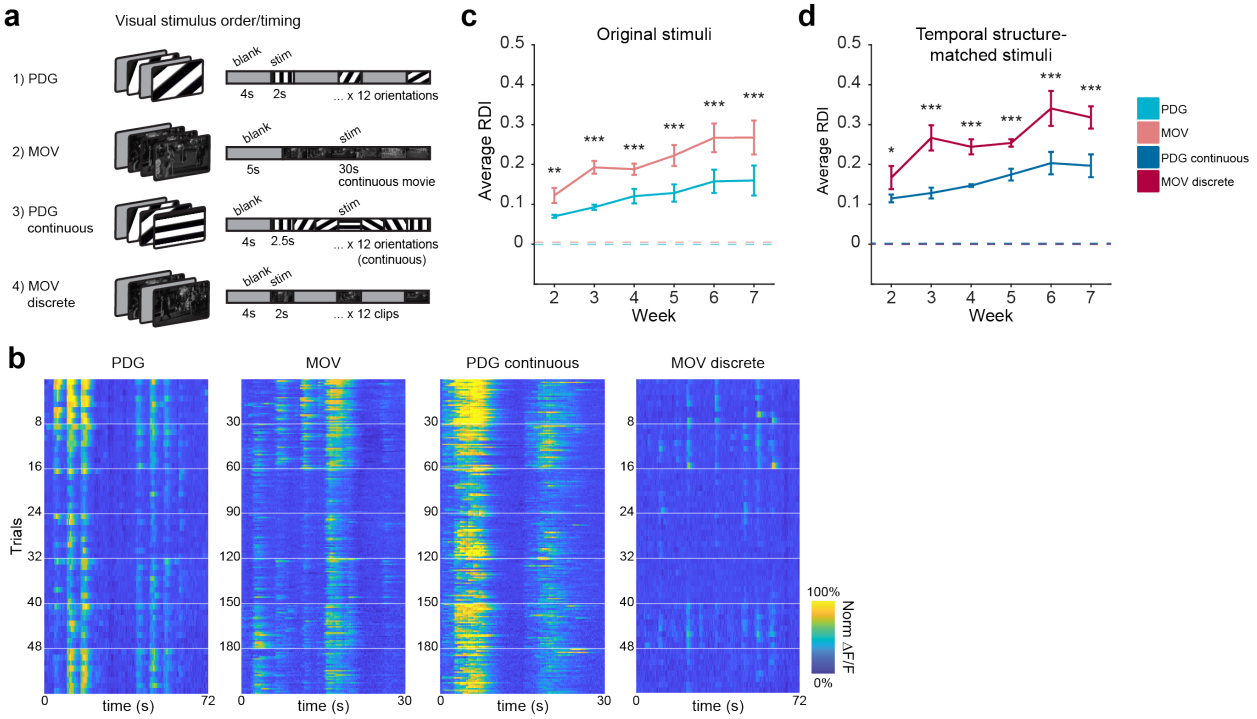

Supplementary Figure 7: Matching temporal structure between stimuli does not eliminate RDI differences.

(a) Schematic of visual stimuli. The PDG continuous stimulus was designed to have similar temporal structure to the MOV stimulus; the MOV discrete stimulus was designed to have similar temporal structure to the PDG stimulus.

(b) Example neuron responses to each of the stimuli. Recording sessions are separated by white lines.

(c) Field-average RDI curves for the original PDG and MOV stimuli (n = 449, 447, 441, 402, 382, 329 neurons in 5, 5, 5, 4, 4, 3 imaging fields for sessions 2-7 respectively). Error bars are ± s.e.m. MOV is significantly different from PDG ($F_{1,898} = 8.7$, $p = 0.003$, $F_{1,894} = 27.0$, $p = 2.6 \times 10^{-7}$, $F_{1,882} = 11.8$, $p = 6.2 \times 10^{-4}$, $F_{1,804} = 13.2$, $p = 3.0 \times 10^{-4}$, $F_{1,764} = 26.8$, $p = 2.9 \times 10^{-7}$, $F_{1,658} = 34.6$, $p = 6.4 \times 10^{-9}$, for sessions 2-7 respectively; two-tailed F-test using a linear mixed-effects model, fixed effect for stimulus, random effect for mouse; $^{**}p < 0.01$, $^{***}p < 0.001$).

(d) Field-average RDI curves for the temporal-structure-matched stimuli (n = 449, 447, 441, 402, 382, 329 neurons in 5, 5, 5, 4, 4, 3 imaging fields for sessions 2-7 respectively). Error bars are ± s.e.m. MOV discrete is significantly different from PDG continuous ($F_{1,898} = 4.8$, $p = 0.03$, $F_{1,894} = 24.9$, $p = 7.1 \times 10^{-7}$, $F_{1,882} = 24.0$, $p = 1.1 \times 10^{-6}$, $F_{1,804} = 18.8$, $p = 1.6 \times 10^{-5}$, $F_{1,764} = 16.2$, $p = 6.2 \times 10^{-5}$, $F_{1,658} = 20.0$, $p = 8.9 \times 10^{-6}$, for sessions 2-7 respectively; two-tailed F-test using a linear mixed-effects model, fixed effect for stimulus, random effect for mouse; $^{*}p < 0.05$, $^{**}p < 0.01$, $^{***}p < 0.001$).

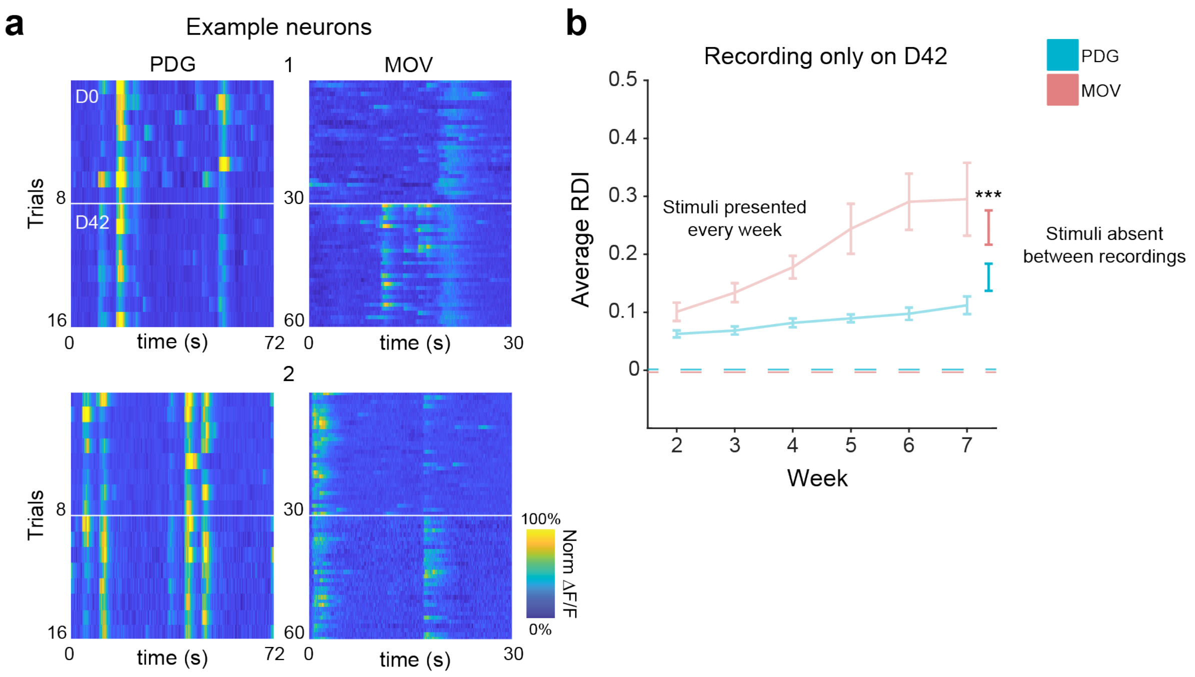

Supplementary Figure 8: Representational drift persists in the absence of repeated stimulus presentation.

(a) Responses to PDG and MOV stimuli on D0 and D42 for two example neurons (top and bottom). White lines separate recording sessions.

(b) Average D42 RDI values (n = 115 neurons from 3 imaging fields) compared to the original average RDI curves from Fig. 1i (desaturated curves). Error bars are ± s.e.m. MOV RDI is significantly different from PDG RDI ($F_{1,228}$ = 11.6, p = 7.4 x 10^{-4}; two-tailed F-test using a linear mixed-effects model, fixed effect for stimulus, random effect for mouse; ***p < 0.001).

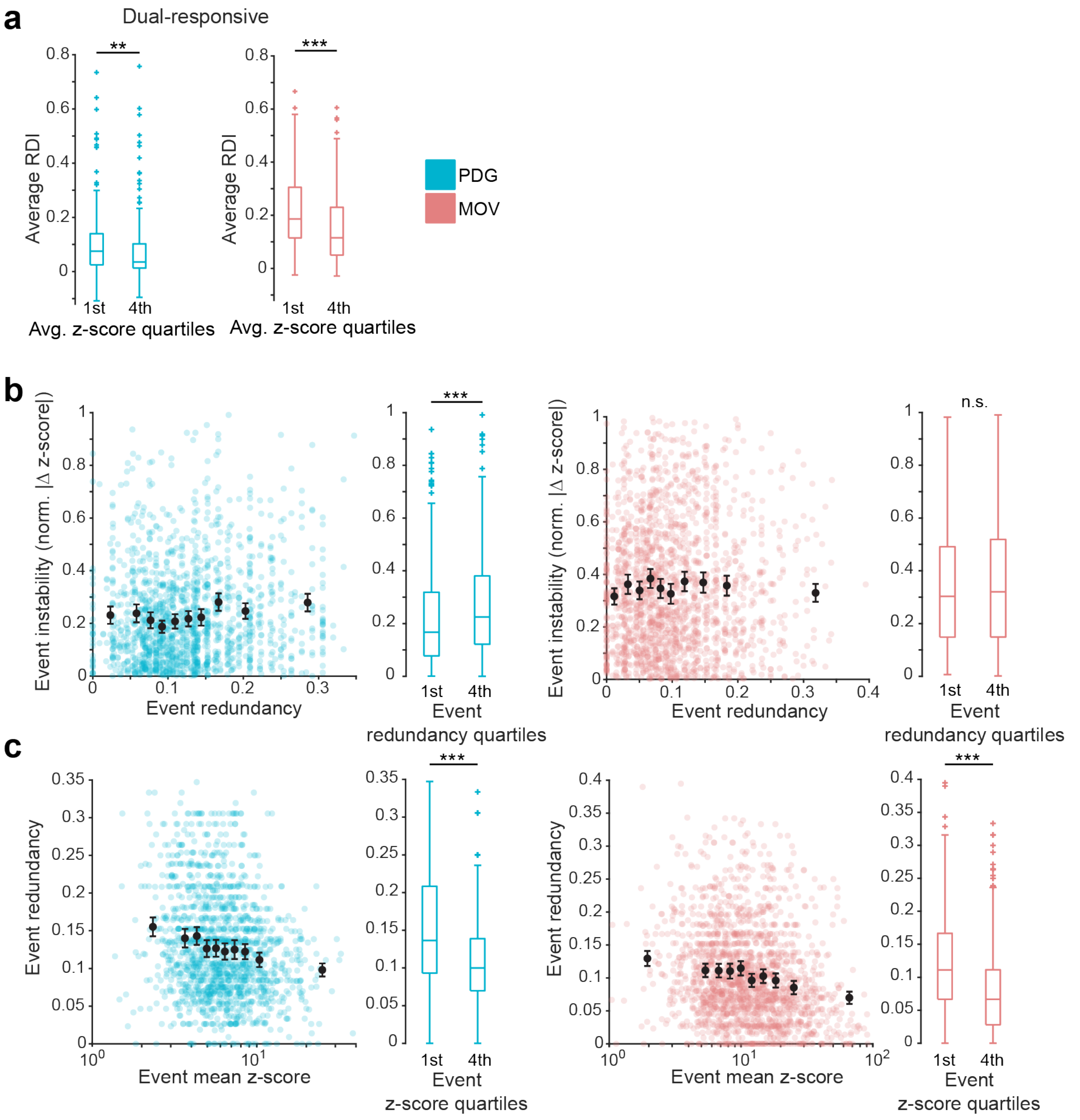

Supplementary Figure 9: Characterization of individual response event stability.

(a) Comparison of first and last quartiles of data from Fig. 2a. Highly active neurons are more stable on average than weakly active neurons. (PDG n = 184 neurons in each quartile, Z = 3.2, p = 1.4 x 10^{-3}; MOV n = 166 neurons in each quartile, Z = 5.1, p = 4.0 x 10^{-7}; two-sided Wilcoxon rank-sum test; **p < 0.01, ***p < 0.001).

(b) Event instability as a function of event redundancy. Redundancy is defined as the fraction of total neurons with at least one event that overlaps at least 75% of a given event's duration. Each colored dot is one event; black dots are 10th percentile binned means ± 95th percent confidence interval. Boxplots compare first and last quartiles of data (PDG n = 383 events in 1st quartile, 369 events in 4th quartile, Z = 3.6, p = 3.8 x 10^{-4}; MOV n = 466 events in 1st quartile, 442 events in 4th quartile, Z = 0.5, p = 0.59; two-sided Wilcoxon rank-sum test; ***p < 0.001). Data shown for all dual-responsive neurons.

(c) Event redundancy as a function of session-average event z-score. Each colored dot is one event; black dots are 10th percentile binned means ± 95th percent confidence interval. Boxplots compare first and last quartiles of data (PDG $n = 365$ events in each quartile, $Z = 7.2$, $p = 5.9 \times 10^{-13}$; MOV $n = 441$ events in each quartile, $Z = 9.1$, $p = 8.7 \times 10^{-20}$; two-sided Wilcoxon rank-sum test; $^{***}p < 0.001$). Data shown for all dual-responsive neurons. All boxplots are centered on median, boxes extend to first and third quartiles, whiskers extend to 1.5 times the interquartile range or minima/maxima in the absence of outliers.

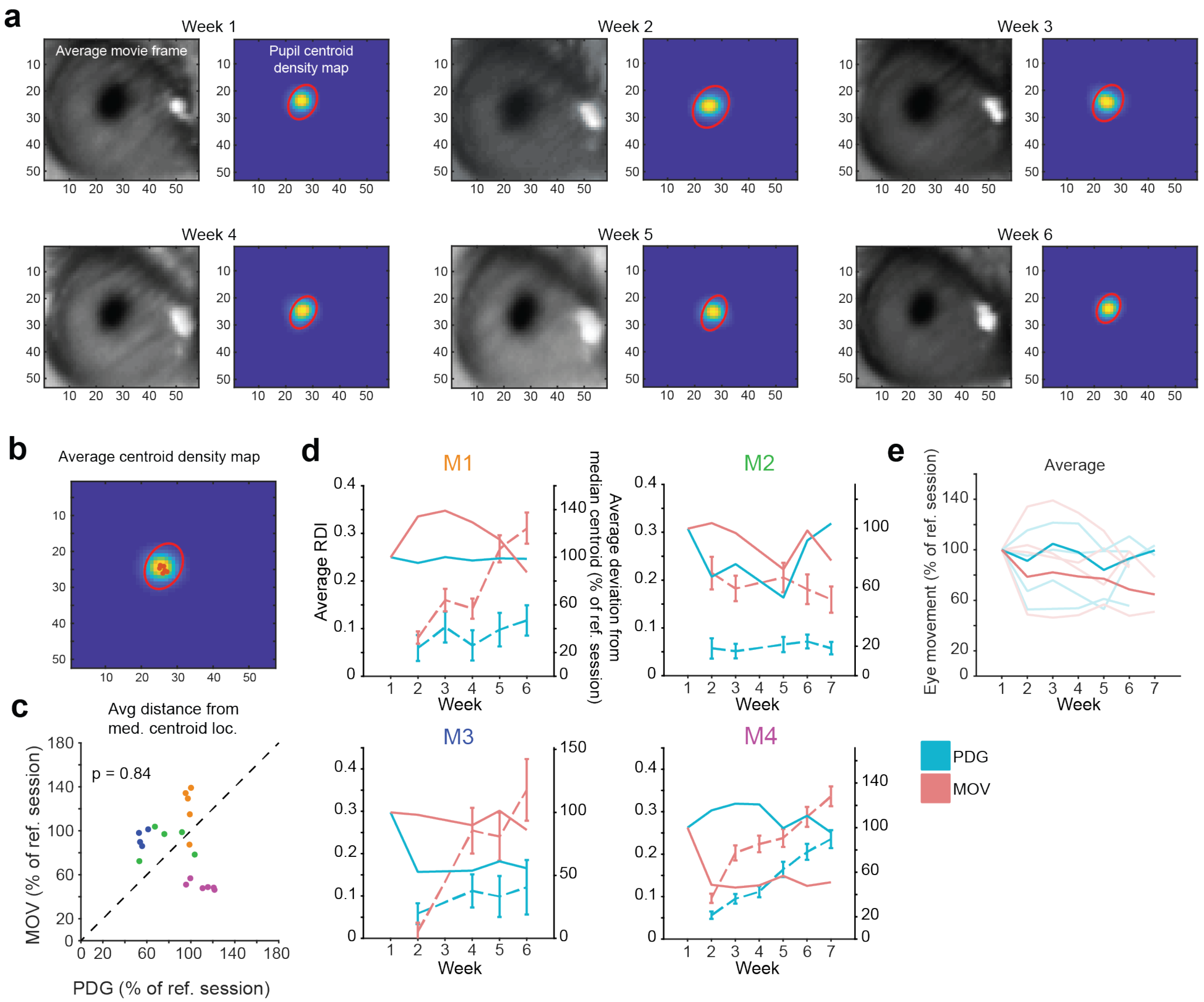

Supplementary Figure 10: Eye movement is minimal and changes in eye movement do not track stability.

(a) Average eye tracking video frames and density heatmaps of pupil centroid location for each recording session. Red ellipse depicts the average size, orientation, and eccentricity of the pupil, centered on the average centroid location.

(b) Session-average pupil centroid density map for example recording in (a). Red ellipse depicts the size, orientation, and eccentricity of the pupil on week 1. Small red circles depict the average pupil centroid location on each recording session.

(c) Between-stimulus comparison of pupil's average distance from median centroid location, expressed relative to the reference session. Each dot is one session, colored by mouse as in (d). Pooled data is not significantly different between stimuli (n = 20 sessions, t_{19} = 0.2, p = 0.84, two-tailed paired-sample t-test).

(d) Eye movement, expressed relative to the reference session (solid lines), and RDI curves (dotted lines are averages across neurons, error bars are ± s.e.m.) for each of the 4 mice tested. RDI for mouse 1: n = 45, 43, 43, 43, 42 neurons for sessions 2-6 respectively; RDI for mouse 2: n = 39, 39, 39, 38, 39 neurons for sessions 2, 3, 5-7 respectively; RDI for mouse 3: n = 17, 17, 16, 15 for sessions 2, 4-6 respectively; RDI for mouse 4: n = 107, 108, 108, 106, 107, 106 neurons for sessions 2-7 respectively.

(e) Pooled and averaged eye movement data across mice.

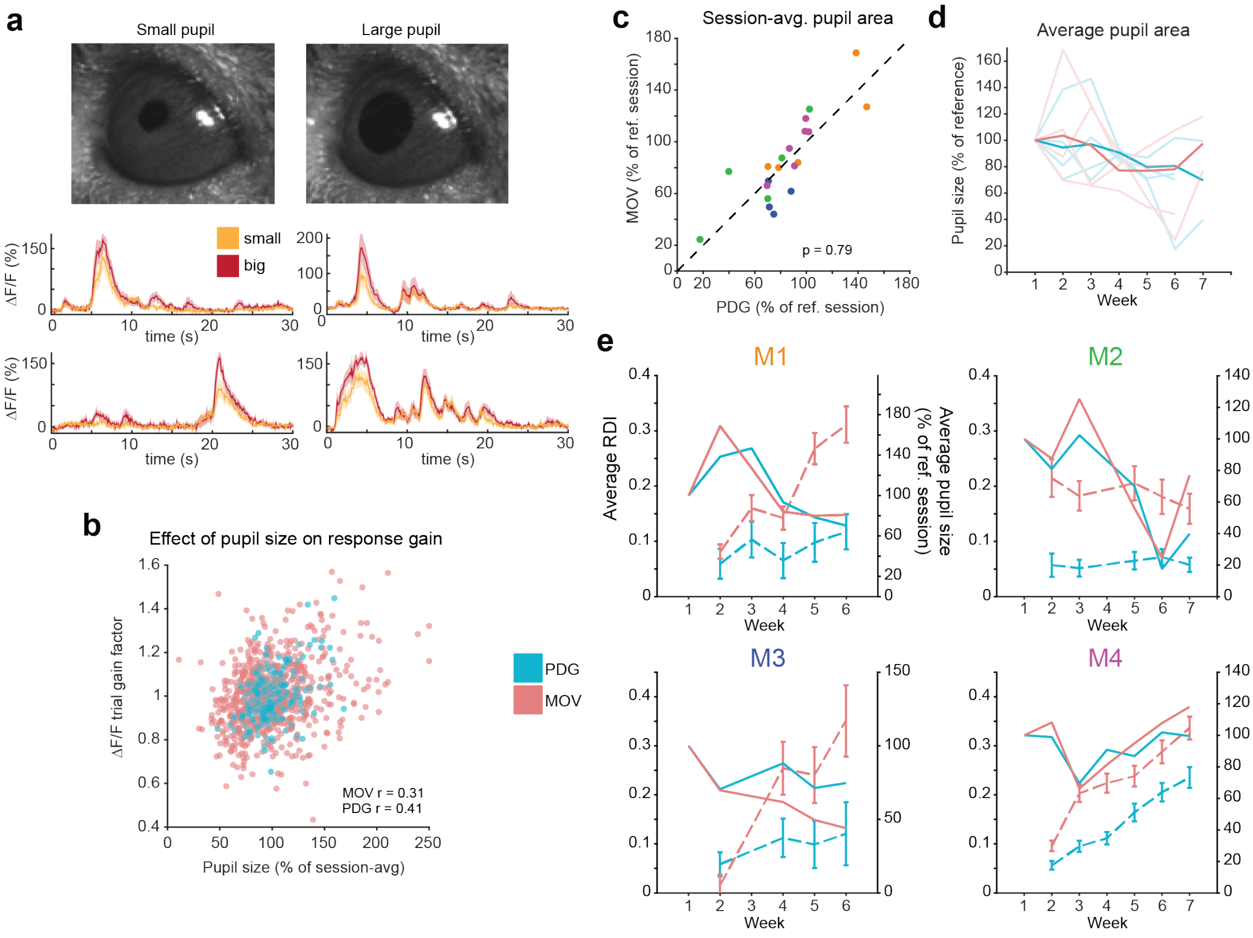

Supplementary Figure 11: Pupil size decreases over time for both stimuli

(a) Top: Images show single frames from trials with small average pupil size and large average pupil size. Bottom: MOV responses to 4 example neurons demonstrating effect of larger pupil size, where orange curves represent average activity across the 10 trials with smallest pupil size and red represents average activity across the 10 trials with greatest pupil size.

(b) Pupil size is correlated with response strength. Each dot represents average pupil size (expressed relative to average pupil size on the session to which the trial belongs) versus response gain on a given trial (MOV: $r = 0.31$, $p = 5.6 \times 10^{-17}$; PDG: $r = 0.41$, $p = 2.5 \times 10^{-9}$; Pearson's correlation coefficient).

(c) Between-stimulus comparison of average pupil size expressed relative to the reference session. Each dot is one session, colored according to each mouse in (e). Pooled data is not significantly different between stimuli ($n = 20$ sessions, $t_{19} = 0.3$, $p = 0.79$, two-tailed paired-sample t-test).

(d) Pooled and averaged pupil size data across mice.

(e) Pupil size, expressed relative to the reference session (solid lines), and RDI curves (dotted lines are averages across neurons, error bars are ± s.e.m.) for each of the 4 mice tested. RDI for mouse 1: n = 45, 43, 43, 43, 42 neurons for sessions 2-6 respectively; RDI for mouse 2: n = 39, 39, 39, 38, 39 neurons for sessions 2, 3, 5-7 respectively; RDI for mouse 3: n = 17, 17, 16, 15 for sessions 2, 4-6 respectively; RDI for mouse 4: n = 107, 108, 108, 106, 107, 106 neurons for sessions 2-7 respectively.

Receptive field shifts, example mice

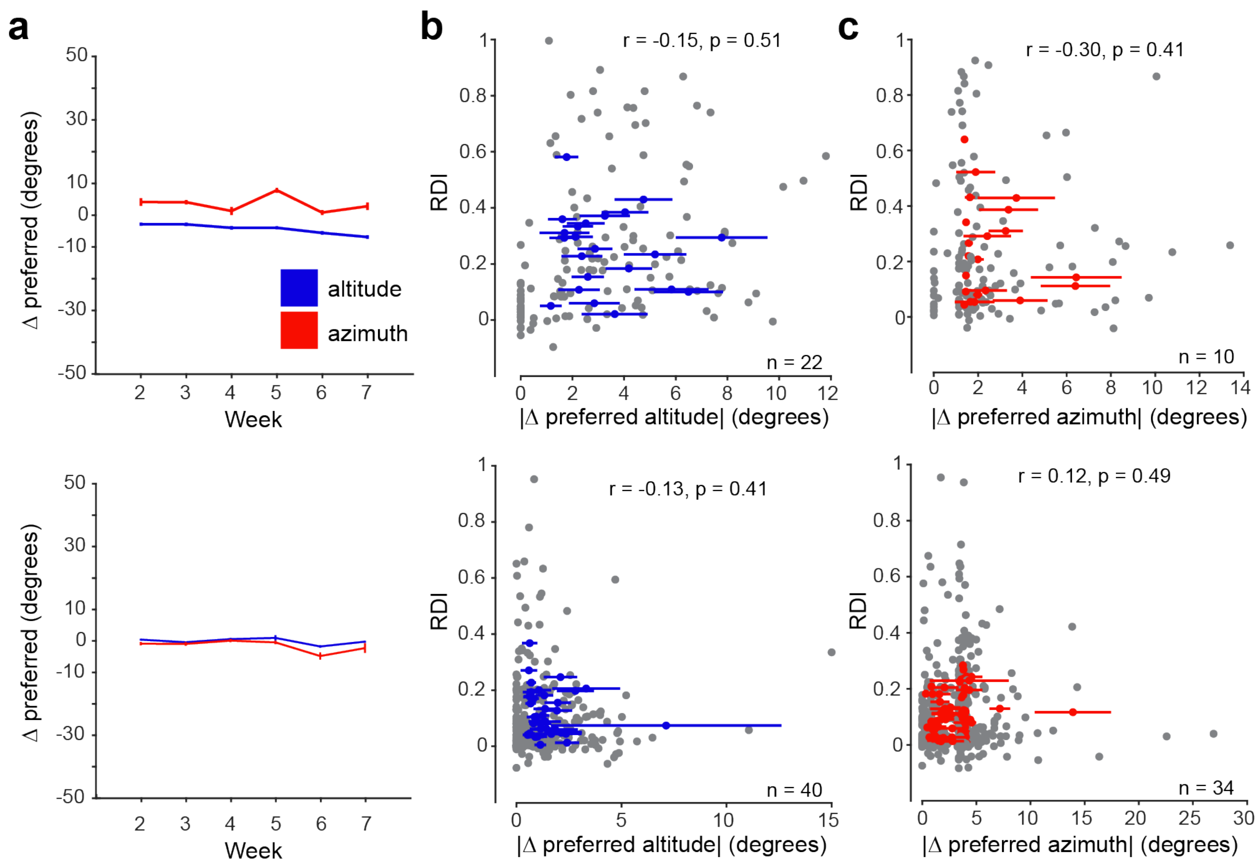

Supplementary Figure 12: RDI is not associated with changes in spatial receptive field location.
(a) Average change in preferred altitude (blue) and azimuth (red) across weeks. Error bars are ± s.e.m. (top example mouse: altitude n = 22 neurons on each session, azimuth n = 10 neurons on each session; bottom example mouse: altitude n = 40 neurons on each session, azimuth n = 34 neurons on each session). The visual stimulus screen spans 100° in the altitude direction and 130° in the azimuth direction.
(b) Effect of a neuron's change in spatial receptive field on its MOV RDI value. Each blue dot is one neuron (n = 22 neurons for top example mouse, top; n = 40 neurons for bottom example mouse), depicting its session-average absolute change in preferred altitude versus its session-average MOV RDI; horizontal error bars are ± s.e.m. Each grey dot is a neuron's absolute change in preferred altitude on a given session versus its MOV RDI value on that session (there are multiple grey dots for every blue dot). Pearson correlation displayed on each plot uses colored data points. Data shown for all well-tracked neurons spatially tuned to altitude and visually responsive to MOV.
(c) Same as in (b), but for preferred azimuth (n = 10 neurons for top example mouse; n = 34 neurons for bottom example mouse). Data shown for all well-tracked neurons spatially tuned to azimuth and visually responsive to MOV.

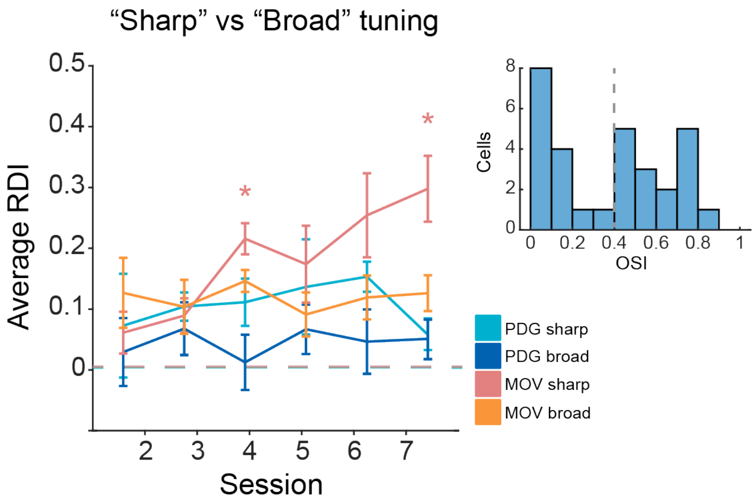

Supplementary Figure 13: Representational drift across stimuli for sharply- and broadly- tuned inhibitory interneurons. RDI over time across stimuli for broadly-tuned neurons (PDG: blue; MOV: orange) and sharply-tuned neurons (PDG: cyan; MOV: pink). Error bars are ± s.e.m. Inset, distribution of OSIs for inhibitory interneurons; grey dashed line in histogram indicates threshold for sharply-tuned (OSI > 0.4) versus broadly-tuned (OSI > 0.4) neurons. Asterisks indicate significant differences for each session for sharp vs. broad within each stimulus (MOV: pink, $F_{1,31} = 0.5$, $p = 0.49$, $F_{1,32} = 0$, $p = 0.94$, $F_{1,32} = 4.3$, $p = 0.047$, $F_{1,31} = 0.2$, $p = 0.65$, $F_{1,31} = 1.1$, $p = 0.30$, $F_{1,29} = 4.2$, $p = 0.049$ for sessions 2-7 respectively; PDG: no sessions show significant difference; $F_{1,31} = 0.1$, $p = 0.71$, $F_{1,32} = 1.5$, $p = 0.23$, $F_{1,32} = 2.4$, $p = 0.13$, $F_{1,31} = 0.4$, $p = 0.53$, $F_{1,31} = 2.5$, $p = 0.12$, $F_{1,29} = 0.2$, $p = 0.67$ for sessions 2-7 respectively; two-tailed F-test using a linear mixed-effects model, fixed effect for stimulus, random effect for mouse; $*p < 0.05$).

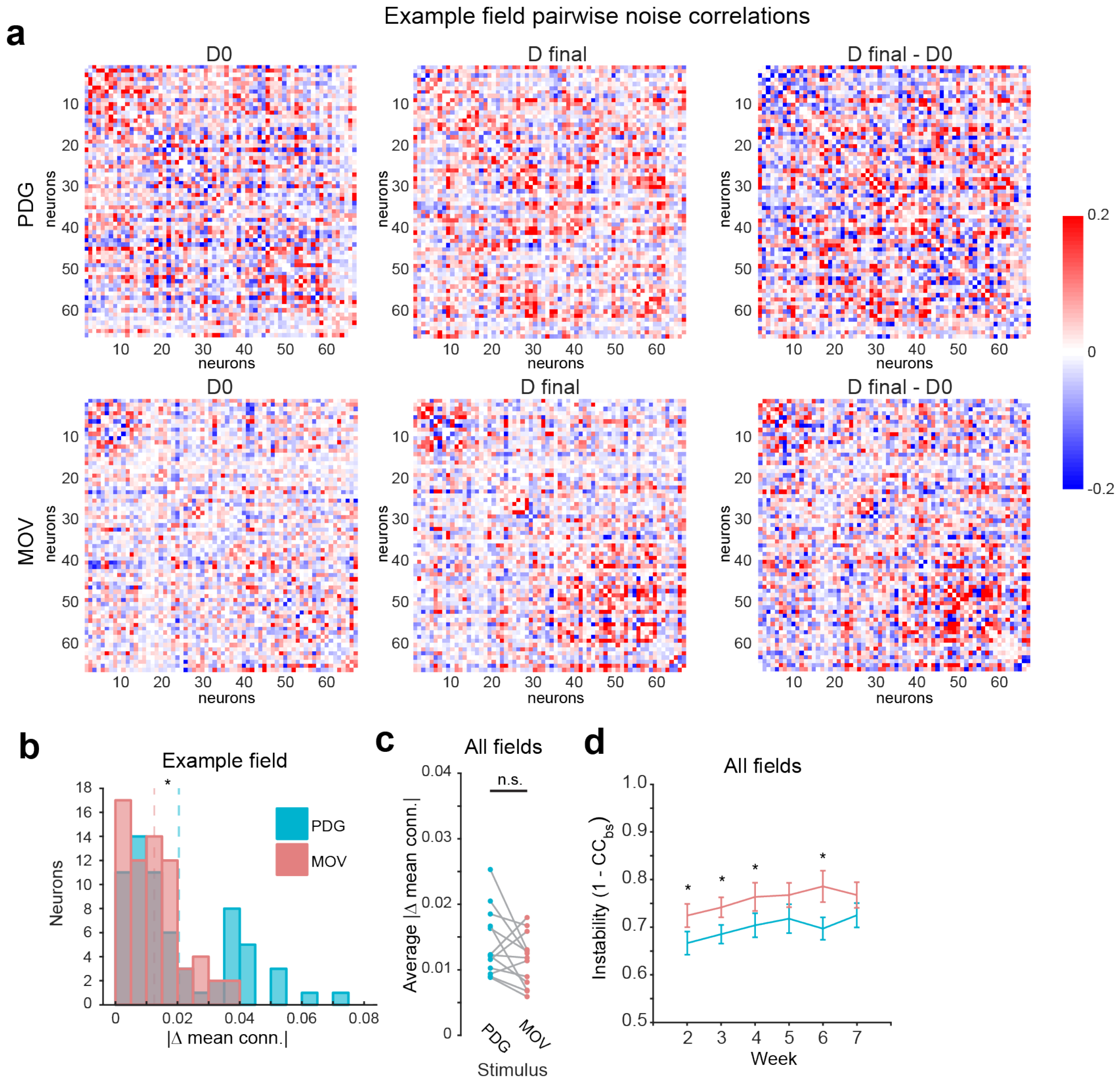

Supplementary Figure 14: Population noise correlation stability is not clearly stimulus-dependent

(a) Pairwise noise correlations on session 1 (left), final session (middle), and their difference (right) for one example field. Neurons are sorted by time of peak response on D0 for each stimulus. Data shown for all neurons responsive to both stimuli.

(b) Distributions of single-neuron average change in noise correlations between first and final sessions for example field in A. Dotted lines are means for each stimulus (n = 66 neurons, Z = 2.5, p = 0.012, two-sided Wilcoxon rank-sum test; *p < 0.05).

(c) Field-average changes in noise correlation between first and final sessions. Data shown for all fields. (n = 13 fields, t_{12} = 1.8, p = 0.92, two-tailed paired-sample t-test). Although the example field in (b) showed differential noise correlation stability between stimuli, there is no consistent difference across fields.

(d) Average instability of noise correlation matrices with respect to the first session over time (1 – CC_{bs}; where CC_{bs} is the 2D cross correlation between noise correlation matrices). Data shown for all fields. Error bars are ± s.e.m.; significance markers indicate comparison of PDG and MOV values on given session (n = 13 fields; t_{12} = 2.7, p = 0.018, t_{11} = 2.3, p = 0.042, t_{11} = 2.2, p = 0.047, t_{12} = 1.5, p = 0.15, t_{10} = 2.4, p = 0.038, t_8 = 2.0, p = 0.08, for sessions 2-7 respectively; two-tailed paired-sample t-test; *p < 0.05).
