# Supplementary material for: Stimulus-dependent representational drift in primary visual cortex
Source: Nat Commun. 2021 Aug 27;12:5169. doi: 10.1038/s41467-021-25436-3 (PMC8397766; doi:10.1038/s41467-021-25436-3)
Supplement: Supplementary file 3 — Reporting Summary [file 41467_2021_25436_MOESM3_ESM.pdf]

Corresponding author(s): Michael Goard
Last updated by author(s): May 25, 2021

Reporting Summary

Nature Research wishes to improve the reproducibility of the work that we publish. This form provides structure for consistency and transparency in reporting. For further information on Nature Research policies, see our Editorial Policies and the Editorial Policy Checklist.

Please do not complete any field with "not applicable" or n/a. Refer to the help text for what text to use if an item is not relevant to your study.
For final submission: please carefully check your responses for accuracy; you will not be able to make changes later.

Statistics

For all statistical analyses, confirm that the following items are present in the figure legend, table legend, main text, or Methods section.

n/a	Confirmed	
☐	☒	The exact sample size (*n*) for each experimental group/condition, given as a discrete number and unit of measurement
☒	☐	A statement on whether measurements were taken from distinct samples or whether the same sample was measured repeatedly
☐	☒	The statistical test(s) used AND whether they are one- or two-sided<br>*Only common tests should be described solely by name; describe more complex techniques in the Methods section.*
☐	☒	A description of all covariates tested
☐	☒	A description of any assumptions or corrections, such as tests of normality and adjustment for multiple comparisons
☐	☒	A full description of the statistical parameters including central tendency (e.g. means) or other basic estimates (e.g. regression coefficient) AND variation (e.g. standard deviation) or associated estimates of uncertainty (e.g. confidence intervals)
☐	☒	For null hypothesis testing, the test statistic (e.g. *F*, *t*, *r*) with confidence intervals, effect sizes, degrees of freedom and *P* value noted<br>*Give P values as exact values whenever suitable.*
☒	☐	For Bayesian analysis, information on the choice of priors and Markov chain Monte Carlo settings
☒	☐	For hierarchical and complex designs, identification of the appropriate level for tests and full reporting of outcomes
☐	☒	Estimates of effect sizes (e.g. Cohen's *d*, Pearson's *r*), indicating how they were calculated

Our web collection on statistics for biologists contains articles on many of the points above.

Software and code

Policy information about availability of computer code

Data collection	All visual stimuli were generated with a Windows PC using MATLAB and the Psychophysics toolbox. For Widefield imaging, images were acquired with pco.edge camera control software and saved into multi-page TIF files. For 2-photon imaging, images were acquired using PrairieView acquisition software (Version 5.4.64.300) and converted into TIF files. All subsequent image processing was performed in MATLAB (Mathworks).
Data analysis	All software used for data processing and analysis was custom MATLAB code.

For manuscripts utilizing custom algorithms or software that are central to the research but not yet described in published literature, software must be made available to editors and reviewers. We strongly encourage code deposition in a community repository (e.g. GitHub). See the Nature Research guidelines for submitting code & software for further information.

Data

Policy information about availability of data

All manuscripts must include a data availability statement. This statement should provide the following information, where applicable:

- Accession codes, unique identifiers, or web links for publicly available datasets
- A list of figures that have associated raw data
- A description of any restrictions on data availability

Most of the hardware designs can be found on our institutional lab website (https://goard.mcdb.ucsb.edu/resources). Neuronal response data from Figures 1-6 are available on Dryad (doi: TBA, final version will be uploaded upon manuscript acceptance). All other raw data are available upon request to corresponding author.

Field-specific reporting

Please select the one below that is the best fit for your research. If you are not sure, read the appropriate sections before making your selection.

☒ Life sciences ☐ Behavioural & social sciences ☐ Ecological, evolutionary & environmental sciences

For a reference copy of the document with all sections, see nature.com/documents/nr-reporting-summary-flat.pdf

Life sciences study design

All studies must disclose on these points even when the disclosure is negative.

Sample size	No a priori sample size calculation was performed, as we did not have an estimate of the effect size. Sample sizes were chosen based on recent studies.
Data exclusions	For individual mice, a given session of data was discarded a priori if the alignment of the imaging field was poor enough such that the registration software was unable to successfully register it with the reference session's data. Mice were excluded a priori if multiple sessions experienced this issue. Individual neurons were excluded from analysis if they did not meet sufficient quality standards as described in our Methods section, were not well-tracked on a given session as determined by visual inspection, or if they did not meet visual responsiveness criteria (dependent on analysis).
Replication	The central finding (RDI difference for PDG and MOV) was replicated in several independent sets of experiments: (1) the initial set of experiments reporting the finding (Figure 1), (2) experiments in which neurons were imaged transcranially (Figure S6), (3) experiments in which the original PDG and MOV stimuli were presented with matching temporal structures (Figure S7), (4) experiments in which mice were only imaged on the first session and last session without intermittent sessions (Figure S8).
Randomization	Animals were not part of distinct experimental groups.
Blinding	Blinding was unnecessary because animals were not part of distinct experimental groups.

Reporting for specific materials, systems and methods

We require information from authors about some types of materials, experimental systems and methods used in many studies. Here, indicate whether each material, system or method listed is relevant to your study. If you are not sure if a list item applies to your research, read the appropriate section before selecting a response.

Materials & experimental systems

n/a	Involved in the study	
☒	☐	Antibodies
☒	☐	Eukaryotic cell lines
☒	☐	Palaeontology and archaeology
☐	☒	Animals and other organisms
☒	☐	Human research participants
☒	☐	Clinical data
☒	☐	Dual use research of concern

Methods

n/a	Involved in the study	
☒	☐	ChIP-seq
☒	☐	Flow cytometry
☒	☐	MRI-based neuroimaging

Animals and other organisms

Policy information about studies involving animals; ARRIVE guidelines recommended for reporting animal research

Laboratory animals	C57BL/6 background laboratory mice, male and female, of ages 12-30 weeks. The following strains were used: (1) Emx1-IRES-Cre (Jax Stock #005628) x ROSA-LNL-tTA (Jax Stock #011008) x TITL-GCaMP6s (Jax Stock #024104). (2) Slc17a7-IRES2-Cre (Jax Stock #023527) x TITL2-GC6s-ICL-TTA2 (Jax Stock #031562). (3) GAD2-IRES-Cre (Jax Stock #028867) x TITL2-GC6s-ICL-TTA2 (Jax Stock #031562).
Wild animals	This study did not involve wild animals.
Field-collected samples	This study did not involve field-collected samples.
Ethics oversight	All animal procedures were approved by the Institutional Animal Care and Use Committee at University of California, Santa Barbara.

Note that full information on the approval of the study protocol must also be provided in the manuscript.

This checklist template is licensed under a Creative Commons Attribution 4.0 International License, which permits use, sharing, adaptation, distribution and reproduction in any medium or format, as long as you give appropriate credit to the original author(s) and the source, provide a link to the Creative Commons license, and indicate if changes were made. The images or other third party material in this article are included in the article's Creative Commons license, unless indicated otherwise in a credit line to the material. If material is not included in the article's Creative Commons license and your intended use is not permitted by statutory regulation or exceeds the permitted use, you will need to obtain permission directly from the copyright holder. To view a copy of this license, visit http://creativecommons.org/licenses/by/4.0/
